# Inferring Drug-Related Diseases Based on Convolutional Neural Network and Gated Recurrent Unit

**DOI:** 10.3390/molecules24152712

**Published:** 2019-07-25

**Authors:** Ping Xuan, Lianfeng Zhao, Tiangang Zhang, Yilin Ye, Yan Zhang

**Affiliations:** 1 School of Computer Science and Technology, Heilongjiang University, Harbin 150080, China; 2 School of Mathematical Science, Heilongjiang University, Harbin 150080, China

**Keywords:** drug-disease association prediction, convolutional neural network, gated recurrent unit, attention mechanism at path level, drug repositioning

## Abstract

Predicting novel uses for drugs using their chemical, pharmacological, and indication information contributes to minimizing costs and development periods. Most previous prediction methods focused on integrating the similarity and association information of drugs and diseases. However, they tended to construct shallow prediction models to predict drug-associated diseases, which make deeply integrating the information difficult. Further, path information between drugs and diseases is important auxiliary information for association prediction, while it is not deeply integrated. We present a deep learning-based method, CGARDP, for predicting drug-related candidate disease indications. CGARDP establishes a feature matrix by exploiting a variety of biological premises related to drugs and diseases. A novel model based on convolutional neural network (CNN) and gated recurrent unit (GRU) is constructed to learn the local and path representations for a drug-disease pair. The CNN-based framework on the left of the model learns the local representation of the drug-disease pair from their feature matrix. As the different paths have discriminative contributions to the drug-disease association prediction, we construct an attention mechanism at the path level to learn the informative paths. In the right part, a GRU-based framework learns the path representation based on path information between the drug and the disease. Cross-validation results indicate that CGARDP performs better than several state-of-the-art methods. Further, CGARDP retrieves more real drug-disease associations in the top part of the prediction result that are of concern to biologists. Case studies on five drugs demonstrate that CGARDP can discover potential drug-related disease indications.

## 1. Introduction

In the past decades, there has been a gradual increase in new molecular entity research and development, but the number of new molecular entities approved by the Food and Drug Administration (FDA) has been decreasing [1,2,3]. Traditional drug development often requires 10–15 years and an investment of $1.5 billion [4,5,6]. Because FDA-approved drugs undergo biological experiments, clinical trials, and are evaluated for safety, drugs are often repositioned. Repositioning existing drugs for new indications or uses requires only 6.5 years, and the cost is $300 million, which is far less than the cost of developing a new drug [7,8,9].

Based on different biological premises and assumptions, researchers use different data types and biological preconditions to study drug repositioning. Research methods include retargeting based on drug targets [10,11], relocation based on drug side effects [12,13,14], and heterogeneity based on drug diseases [15,16,17,18]. Most drug targets are directly linked to the pathogenesis of the diseases. Li et al. constructed a drug-target heterogeneous network using similarities between the targets and the drugs to integrate information between the target and the drug for drug repositioning. Zhao et al. [19] used target gene information and disease-causing gene information to calculate drug similarities and disease similarities, and they finally identified a gene-disease relationship through the Bayesian method. Wang et al. [20] proposed a three-layer heterogeneous network that integrates drug similarities, disease similarities, drug-disease associations, and drug-target interactions to disseminate information for predicting the relationship between the drugs and the diseases. However, drugs can cause off-target phenomena in the living environment and produce unexpected side effects; therefore, drug side effects are also one of the essential factors for repositioning drugs. Campillos et al. [16] proposed a drug side effect similarity to determine whether two drugs are involved in the same target. Gottlieb et al. [21] and Zhang et al. [22] used drug chemical substructure, side effects, etc. to calculate drug similarities using logistic regression and collaborative filtering algorithms to predict potential drug-diseases relationship. However, these methods are not suitable for drugs and diseases that do not have a common gene or target.

Most advanced methods are predictive for drug–disease networks. Liang et al. [23] used drug chemical substructure information, drug target domain information, and drug target annotation information to calculate drug similarities; the drug-disease associations were predicted through Laplacian regularized sparse subspace learning (LRSSL). Luo et al. [24] used drug chemical substructure information to calculate drug similarities, and they used disease semantics to calculate disease similarities. Then, they constructed a drug-disease two-layer heterogeneous network using a bi-random walk with a restart algorithm to reposition drugs. Zhang et al. [25] also used drug similarity and disease similarity to design drug–disease heterogeneous networks for repositioning drugs based on matrix decomposition with similarity constraints. Xuan et al. [26] proposed a matrix-based decomposition method for integrating drug similarity and disease similarity to predict drug–disease associations. However, these methods are shallow learning methods that cannot accommodate complex and non-linear information on drug similarity, disease similarity, and drug–disease associations. In addition, the paths of drugs and diseases as important auxiliary information were not deeply integrated in these previous methods. Therefore, a deep-learning-based prediction method must be developed to integrate the similarity, association information, and path information of drug–disease pairs. We propose a prediction method based on a convolutional neural network (CNN) and gated recurrent unit (GRU) called CGARDP for predicting drug-disease associations. The left part of CGARDP’s prediction model focuses on local information related to a drug-disease pair, and the right part of the model learns the path information between drug-disease pairs. Experimental cross-validation results clearly show that CGARDP performs better than several of the most advanced prediction methods. Case studies involving five drugs show that CGARDP can detect potential candidate disease indications.

## 2. Materials and Methods

### 2.1. Dataset

We obtained drug-disease association data from study [26], which involved 763 drugs and 681 diseases. The chemical fingerprints extracted from the PubChem database [27] were used for representing the chemical substructures of drugs. Disease information can be obtained from the MeSH database [28]. We obtained drug similarity and disease similarity data from a work published on LRSSL [23].

### 2.2. Construction of Drug-Disease Network

The more similar the chemical substructures of two drugs are, the more likely the drugs are to act on similar functions. The chemical substructure vector $S_{i}$ of a drug $r_{i}$ is an 869-dimensional binary vector. We defined $S_{i}=\{sub_{i,1},sub_{i,2},\ldots ,sub_{i,j},\ldots ,sub_{i,869}\}$, where $sub_{i,j}$ is the *j*-th chemical substructure of the *i*-th drug. LRSSL [23] measured the drug similarities by calculating the cosine similarities between the chemical substructures of drugs. We also use $R=R_{\left[i,j\right]}\in R^{N_{r}\times N_{r}},$ which represents drug similarity, where $R_{\left[i,j\right]}$ is in the range of [0, 1] and is the similarity of $r_{i}$ and $r_{j}$, and $N_{r}$ denotes the number of drugs. 

To evaluate the similarity between diseases, we establish directed acyclic graphs (DAG) of semantic terms for corresponding diseases, which contain all semantic terms related to that disease. Wang et al. [28] successfully calculated the semantic similarity between diseases using their related terms in the DAG graph. LRSSL computed the similarities between diseases by using Wang’s method, and we obtained the disease similarity from LRSSL. Let $D=D_{\left[i,j\right]}\in R^{N_{d}\times N_{d}}$ be a similarity matrix of diseases such that each element is between 0 and 1.

**Figure 1:**
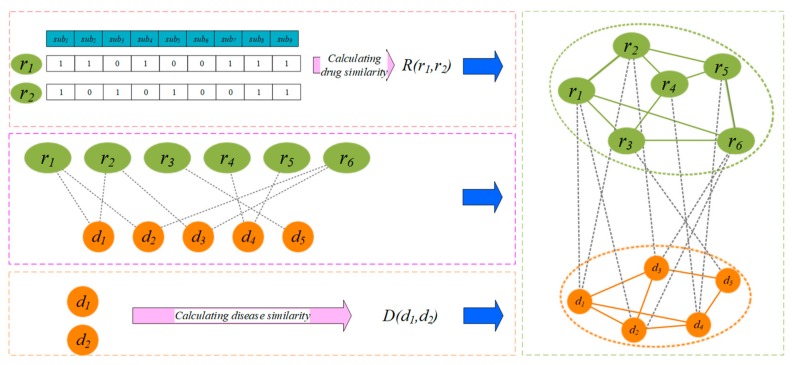
Construction of a drug-disease heterogeneous network based on the similarity calculation.

In light of the relationship between drugs and diseases, we add an edge between the corresponding drug and disease (Figure 1). Matrix $A\in R^{N_{r}\times N_{d}}$ denotes the edge set; if $A_{ij}=1$, drug $r_{i}$ is associated with the disease $d_{j}$, otherwise,$\;A_{ij}=0$.

### 2.3. Prediction Model Based on CNN and GRU

To predict the potential representation of the association between a drug and a disease, we propose a novel prediction model based on a CNN and GRU. We apply the CNN module in the left part to learn the combinatorial representation of drug $r_{i}$ and disease $d_{j}$; further, we apply GRU in the right part to capture the path representation between $r_{i}$ and $d_{j}$. Finally, the two representations were integrated by a combined strategy to achieve the final correlation scores of $r_{i}$ and $d_{j}$. We take drug $r_{1}$ and disease $d_{3}$ as an example to describe the learning framework for the left and right parts, and we use *x,* **x, X** to represent the scalar, vector, and matrix, respectively.

**Figure 2:**
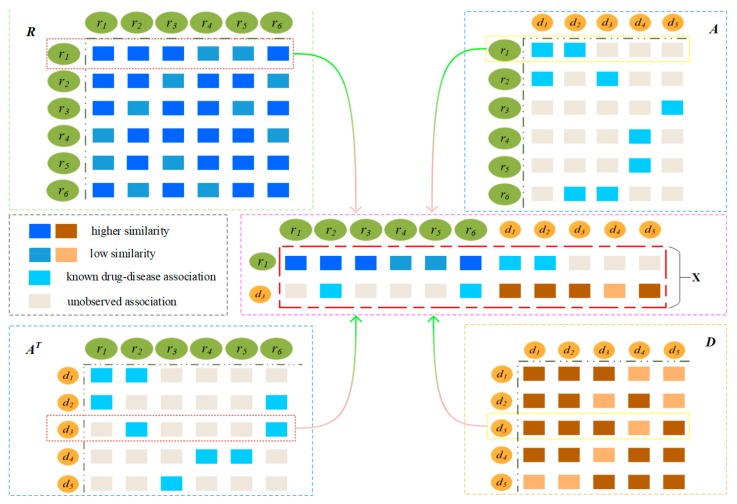
Construction of the feature matrix by integrating the similarities and associations.

The probability that a drug is associated with a disease is higher when there are more drugs similar to another drug associated with a disease, such as $r_{1}$ and $d_{3}$. As shown in Figure 2, drugs similar to $r_{1}$ are {$r_{2},\;r_{3},\;r_{6}$}, and the drugs associated with $d_{3}$ are {$r_{2},r_{6}$}. The drugs associated with $d_{3}$ are similar to $r_{1}$, and therefore, the probability of $d_{3}$ being associated with $r_{1}$ is very high. The first row of matrix **R** denotes the similarity between $r_{1}$ and all drugs, and the third row of the matrix $A^{T}$ denotes as the associations between $d_{3}$ and all drugs.

A drug is associated with more diseases that are similar to a disease, so the more likely the drug is associated with the disease, such as $r_{1}$ and $d_{3}$. As shown in Figure 2, diseases similar to $d_{3}$ are {$d_{1},d_{2},d_{5}$} and the $r_{1}$ associated with $\left\{d_{1},d_{2}\right\}$; therefore, $r_{1}$ and $d_{3}$ are more likely to be related. The third row of the matrix **D** denotes the similarity between $d_{3}$ and all diseases, and the first row of matrix **A** denotes the association between $r_{1}$ and all diseases.

Therefore, we combine the left and right feature representations into the feature matrix $X=X_{\left[i,j\right]}\in R^{2\times (N_{r}+N_{d})}$ of $r_{1}$ and $d_{3}$, $N_{r}$ is the number of drugs and $N_{d}$ is the number of diseases. The first row of the matrix **X** denotes the eigenvector of drug $r_{1}$, and the second row denotes the eigenvector of disease $d_{3}$.

#### 2.3.1. Convolution Module on the Left

##### Convolutional Layer

**Figure 3:**
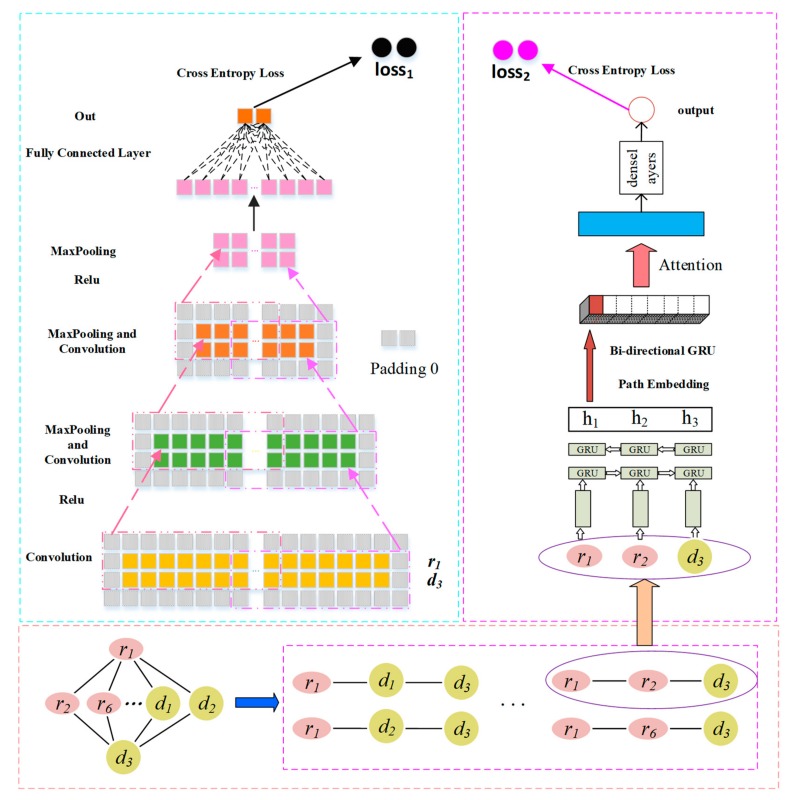
Drug-disease association prediction framework based on convolutional neural network (CNN) and gated recurrent unit (GRU).

As shown in Figure 3, to capture the boundary information of X, we first apply a padding operation obtain a new matrix named $X^{\prime }$. Then, we use $X^{\prime }$ as an input to the left convolution module [29] to learn the potential representation of a drug-disease pair. We assume that the size of the filter is set as $W_{f}$ and $W_{h}$ for each layer of convolution. When there are $n_{conv}$ filters, the convolution filter $W_{conv}\in R^{W_{f}\times W_{h}\times n_{conv}}$ is applied to $X^{\prime }$. Then, we obtain the feature matrix $Z_{conv}\in R^{\left(2-W_{h}+2p+1\right)\times \left(d-W_{f}+2p+1\right)\times n_{conv}}$, where *p* is the number of padding layer in the feature matrix of the CNN model, and *d* is the length of $X^{\prime }$. $X_{conv}^{\prime }\left(i,j\right)$ is the element at the *i*-th row and the *j*-th column of $X^{\prime }$, and $X_{conv}^{\prime }\left(k,i,j\right)$ represents a region within the filter when the *k*-th filter slides to the $X_{conv}^{\prime }\left(i,j\right)$. The formal definitions of $X_{conv}^{\prime }\left(k,i,\;j\right)$ and $Z_{conv,k}\left(i,j\right)$ are as follows:

(1) $$X_{conv}^{\prime }\left(k,i,j\right)=X_{conv}^{\prime }\left(i:i+w_{f},\;j:j+\;w_{h}\right),X_{conv}^{\prime }\in R^{W_{f}\times W_{h}},$$

(2) $$Z_{conv,k}i,j=\;f\left(X_{conv}^{\prime }\left(k,i,\;j\right)*W_{conv}\left(k,:,:\right)+b_{conv}\left(k\right)\right),$$

(3) $$i\in \left[1,2-W_{h}+2p+1\right],\;j\in \left[1,d-W_{f}+2p+1\right],\;k\in \left[1,n_{conv}\right],$$

where $W_{conv}\left(k,:,:\right)$ is the sliding window weight matrix of the k-th filter, $b_{conv}$ is the bias vector, f is a ReLU function [30], $Z_{conv,k}i,j$ is the element at the i-th row and j-th column of the k-th feature map $Z_{conv,k}$.

##### Pooling Layer

The feature maps $Z_{conv,k}$ are pooling layers for downsampling to remove unimportant sample data, thus further reducing the number of parameters. We use max pooling to complete the pooling operation and set its sampling window size to $W_{m}\times W_{p}$. The pooling outputs of all the feature maps are $Z_{convpool,k}$:

(4) $$Z_{convpool,k}\left(i,j\right)=\mathrm{Max}\left(Z_{conv,k}\left(i:i+W_{m},j:j+W_{p}\right)\right),$$

(5) $$i\in \left[1,\;2-W_{m}+2p+1\right],\;j\in \left[1,d-W_{p}+2p+1\right],\;k\in \left[1,n_{conv}\right],$$

where $Z_{convpool,k}$ is the *k*-th feature map, and $Z_{convpool,k}\left(i,j\right)$ is the element at its’ *i*-th row and *j*-th column, and *p* is the number of padding layer in the $Z_{conv,k}$. We obtain the feature representation of the node pair $Z_{convpool,k}\left(i,j\right)$, which is flattened and sent to the fully connected layer. The characteristic of the output represents the final result obtained by flattening the fully connected layer as a potential association for the final drug–disease pair *c*:

(6) $$c=\sigma (Z_{convpool,k}\cdot W_{l}),$$

(7) $$W_{l}\in R^{\left(\frac{2-W_{h}+2p}{S}+1\right)\times \left(\frac{d-W_{f}+2p}{S}+1\right)\times 2},$$

where σ is a sigmoid function [31], $W_{l}$ is a fully connected layer feature matrix, and · is the dot product symbol.

#### 2.3.2. GRU with Attention-Based Path Encoder on the Right

For the prediction of the novel association between drug $r_{i}$ and disease $d_{j}$, the different paths between the two nodes contribute differently to their associations. Thus, a path-level attention mechanism is introduced to select more important paths for the association between $r_{i}$ and $d_{j}$. This mechanism consists of two parts: a path encoder and a path attention layer, as shown in Figure 3.

##### GRU-Based Sequence Encoder

The GRU module [32] tracks the state of paths with a gating mechanism instead of using separate memory cells. There are two types of gates: the reset gate $r_{t}$ and the update gate $z_{t}$. These gates jointly control the amount of information that is updated to the state. To illustrate the updated process of the state, we take $r_{1}$ and $d_{3}$ as an example. There are four paths between $r_{1}$ and $d_{3}$ to form a set $P_{13}=\left\{r_{1}\to r_{2}\to d_{3},r_{1}\to r_{6}\to d_{3},r_{1}\to d_{1}\to d_{3},r_{1}\to d_{2}\to d_{3}\right\}$. The node in each path inputs its corresponding feature vector $x_{t}$. The i-th path in $P_{13}$ is represented by $P_{13}^{i}$, and the new state $h_{t}$ of the *t*-th node is calculated as:

(8) $$h_{t}=\left(1-z_{t}\right)\cdot h_{t-1}+z_{t}\cdot {\tilde{h}}_{t},$$

where $h_{t-1}$ is the state of the t−1 state in the path, and ${\tilde{h}}_{t}$ is the candidate state of the current node. This is a linear interpolation between the previous state $h_{t-1}$ and the current new state ${\tilde{h}}_{t}$ computed with new information. The update gate $z_{t}$ controls the extent to which the previous node information is introduced into the current state. The closer the gate $z_{t}$ is to 1, the more the state information of the previous node is brought in. $z_{t}$ is updated as:

(9) $$z_{t}=\sigma \left(W_{z}x_{t}+U_{z}h_{t-1}+b_{z}\right),$$

where $x_{t}$ is the vector at the *t*-th node, $W_{z}$ is the weight matrix of the node vector, $U_{z}$ is the weight matrix of the previous state, and $b_{z}$ is a bias vector. The candidate state ${\tilde{h}}_{t}$ is calculated as:

(10) $${\tilde{h}}_{t}=\tan h\left(W_{h}x_{t}+r_{t}\cdot (U_{h}h_{t-1})+b_{h}\right),$$

where $r_{t}$ is the reset gate that controls how much the past state contributes to the candidate state. If $r_{t}$ is zero, it will forget all previous states.$\;W_{h}$ and $U_{h}$ are matrices of the candidate state, $b_{h}$ is the bias vector of the candidate state, and · is the Hadamard product symbol. The reset gate is updated as:

(11) $$r_{t}=\sigma \left(W_{r}x_{t}+U_{r}h_{t-1}+b_{r}\right),$$

where σ is the sigmod function, $W_{r}$ is the weight matrix of the node vector $x_{t}$ in the reset gate, $U_{r}$ is the weight matrix of the candidate state $h_{t-1}$, and $b_{r}$ is the bias vector.

##### GRU-Based Path Encoder

We assume that $P_{ij}^{t}$ is the path set of drug $r_{i}$ and disease $d_{j}$, and the *t*-th path contains nodes. We use a bidirectional GRU module to integrate the information in two directions of the path and combine the context information of the path nodes. A bidirectional GRU module contains a forward $\vec{GRU}$ module, which reads from the first node to the last node, and the backward $\overset{\leftarrow }{GRU}$ module, which reads from the last node to the first node as:

(12) $$\vec{h_{ij}^{t}}=\vec{GRU}\left(P_{ij}^{t}\right),$$

(13) $$\overset{\leftarrow }{h_{ij}^{t}}=\overset{\leftarrow }{GRU}\left(P_{ij}^{t}\right).$$

we concatenate $h_{ij}^{t}$ and $h_{ij}^{t}$ to obtain the representation $h_{ij}^{t}=[\vec{h_{ij}},\overset{\leftarrow }{h_{ij}}]$ of the *t*-th path of $r_{i}$ and $d_{j}$.

##### Path Attention

To distinguish the different contributions of multiple paths from $r_{i}$ to $d_{j}$ to their associated predictions, we introduce attention mechanisms to distinguish the importance of the path. The total path information $g_{ij}$ is formulated as the weighted sum of all paths, and it is expressed as:

(14) $$g_{ij}=\sum \alpha_{ij}^{t}h_{ij}^{t},$$

where $h_{ij}^{t}$ is the representation vector of the *t*-th path of $r_{i}$ to $d_{j}$, and $\alpha_{ij}^{t}$ is the attention weight of $h_{ij}^{t}$ to measure the importance of the *t*-th path. We introduce a path vector $u_{p}$ to measure the importance of the path. The attention weight of each path can be defined as:

(15) $$u_{ij}^{t}=\tan h\left(W_{t}h_{ij}^{t}+b_{t}\right),$$

(16) $$\alpha_{ij}^{t}=\frac{\exp\left({\left(u_{p}\right)}^{T}u_{ij}^{t}\right)}{\sum_{t}\exp\left({\left(u_{p}\right)}^{T}u_{ij}^{t}\right)},$$

where $u_{ij}^{t}$ is the score function of the corresponding path, i.e., the score of the import of the path, $W_{t}$ is the weight vector, $b_{t}$ is the bias vector,$\;\alpha_{ij}^{t}$ is the attention weight of the *t*-th path, $u_{p}$ is the weight vector, and ${\left(u_{p}\right)}^{T}$ indicated its transposition.

#### 2.3.3. Combined Strategy

To fully combine the representation of the left-path node pair $r_{1}$ and $d_{3}$ and path information representation of the right path, we design a combined strategy for determining the association score of $r_{1}$ and $d_{3}$. We added a SoftMax classifier to ensure that left and right paths have certain predictive capabilities and to further improve the performance of predictive classification. The corresponding loss is defined as:

(17) $${\mathrm{score}}_{c}=\mathrm{softmax}\left(W_{c}c_{ij}+b_{c}\right),$$

(18) $$loss_{1}=y_{real}logscore_{c}^{0}+\left(1-y_{real}\right)\log score_{c}^{1},$$

(19) $${\mathrm{score}}_{g}=\mathrm{softmax}\left(W_{v}g_{ij}+b_{v}\right),$$

(20) $$loss_{2}=y_{real}logscore_{g}^{0}+\left(1-y_{real}\right)\log score_{c}^{1},$$

where $c_{ij}$ is a representational learning method based on CNN learning drug $r_{i}$ and disease $d_{j}$. $g_{ij}$ is the representation obtained by learning on the right, $W_{c}$ and $W_{v}$ are the weight matrices of the left and right parts, respectively, $b_{c}$ and $b_{v}$ are the offset vectors, $y_{real}$ is the actual correlation between the drug and the disease. Further, 1 means the drug is associated with the disease, and 0 is the unknown association, where $score_{c}^{0}$ indicates that there is no possibility of association between drug $r_{i}$ and disease $d_{j}$, and $\;score_{c}^{1}$ indicates that there is no possibility of association between drug $r_{i}$ and disease $d_{j}$. Finally, $loss_{1}$ and $loss_{2}$, are the cross entropy losses of the model in the probability of prediction and the true correlation value. The final loss function of our model is the weighted sum of $loss_{1}$ and $loss_{2}$:

(21) $$\mathrm{loss}=\alpha_{1}loss_{1}+\left(1-\alpha_{1}\right)loss_{2}.$$

where $\alpha_{1}$ is a super parameter, which is used to weigh the contribution of $loss_{1}$ and $loss_{2}$. Our final score is:

(22) $$\mathrm{score}=\alpha_{1}score_{c}+(1-\alpha_{1})score_{g}.$$

#### 2.3.4. Reducing Overfitting

Our neural network has nearly 50 million parameters, which turns out to too many parameters to learn without considerable overfitting. Thus, we introduce the following measures to prevent overfitting.

##### Dropout

Integrating the result from many different models is an excellent method to reduce test errors [33,34], but this method is too computationally expensive for large neural networks and takes several days to train. There is, however, a very efficient approach to model combination that only spends a factor of about two during training. The recently presented technique, called “dropout” [35], consists of setting the output of each hidden neuron to zero with probability 0.5. The neurons that are “dropped out” in this way do not participate in the forward pass and back-propagation. Thus, every time an input is presented, the neural network samples a different architecture, but all these architectures share weights. This technique reduces intricate co-adaptations of neurons, because a neuron cannot depend on the existence of other specific neurons. Therefore, it is forced to learn more robust, beneficial features in conjunction with many different random subsets of the other neurons. During the test, we multiply the output of all the neurons by 0.5, which reasonably approximates the geometric mean of the predictive distributions produced by the exponentially many dropout networks.

## 3. Results and Discussion

### 3.1. Evaluation Metrics

In this study, we applied five-fold cross-validation analysis to evaluate the performance of our method. All known drug-disease associations were treated as positive samples and divided randomly into five equal positive subsets. At the same time, unknown associations with a matching number were randomly selected and divided into five negative subsets. In each fold, four positive subsets and four negative subsets were selected for training and the remaining were used to testing. We trained the prediction model based on known associations in the training set and predicted associations in the testing set. Training and testing were repeated five times, and the average of the performance was adopted. In addition, we calculated the drug similarity each time we selected four positive samples. Then, the testing set for each drug was ranked; the higher the candidate disease ranked, the greater was the possibility of association between the drug and the disease.

The CGARDP model was used to obtain the test scores of the associations in the testing set. The scores were ranked in the descending order of the scores, given a threshold *θ*. If the scores were higher than *θ*, they were considered as positive samples, and those below *θ* were considered as negative samples. We calculate different true positive rates (TPRs), false positive rates (FPRs), accuracy (precisions), and recall (recall) in each *θ* as follows

(23) $$TPR=\frac{TP}{TP+FN},FPR=\frac{FP}{TN+FP},$$

(24) $$precision=\frac{TP}{TP+FP},recall=\frac{TP}{TP+FN}$$

where TP indicates the correct identification of the number of positive samples, TN indicates the correct identification of the number of negative samples, FP indicates the number of samples that will be predicted as a positive example, and FN indicates the number of samples identified as a negative sample. Thus, the receiver operating characteristic (ROC) curve [36] can be drawn using different TPRs and FPRs under different *θ*. The area under the curve (AUC) is called the drug-related AUC value. The average AUC of all drugs was used to assess the overall performance of our method. Because the ratio of positive and negative samples is 1:169, there is a large class imbalance. The class imbalance problem is concerned with positive cases, while the two indicators of the PR curve are focused on positive samples; therefore, the PR curve has more credibility than the ROC curve [1]. Thus, we used the PR curve to measure the performance at the same time. Precision is defined as the percentage of real samples that are determined as positive samples, and recall as the percentage of true samples to the total number of actual positive samples.

In addition, biologists always choose to arrange higher-ranking candidate diseases for biological verifications, and therefore, the top of the ranking candidate list must have more positive samples. Therefore, we made another evaluation criterion a performance metric, i.e., we calculated the average recall rate of top-*k* (*k* = 30, 60, 90, 120…). The higher the recall rate, the higher is the proportion of drug-related diseases that are correctly retrieved; further, the better the predictive performance, the higher is the positive sample that is successfully identified.

### 3.2. Comparison with Other Methods

To evaluate the performance of the CGARDP model, we compared it with several state-of-the-art methods including HGBI [37], MBIRW [24], LRSSL [23], and SCMFDD [25]. HGBI builds a three-layer heterogeneous network that uses a combination of drug, disease, and target for prediction. MBIRW builds a two-layer network of drugs and diseases to complete the drug reposition by walking among the drug-disease network. LRSSL, a Laplacian regularized sparse subspace learning method, combines the chemical substructure of the drug, the target domain, and the target annotation for prediction. SCMFDD calculates the Jaccard similarity of the chemical substructure of the drug and the semantic similarity of the disease to predict novel drug-disease association using matrix factorization.

For CGARDP and several other comparison methods, each method must adjust the parameters involved to optimize the prediction performance. In our method, the left convolutional neural network active windows $W_{f}$ and $W_{h}$ are 3 and 20, respectively. It has two convolutional layers; the first of contains 16 convolution kernels, and the second contains 32 convolution kernels, that is, $n_{conv}$ is 16 and 32. The padding parameter P is (1,10). The size of the sampling window ($W_{m}$,$W_{p}$) is set to (2,2), and the super participation $\alpha_{1}$ is 2. For fairness, the parameters of other methods are based on the parameters recommended in the corresponding literature (α=0.4 for HGBI, α=0.3 for MBIRW, μ=0.01,λ=0.01 for LRSSL, $\mu =2^{0},\lambda =2^{2}$ for SCMFDD).

**Figure 4:**
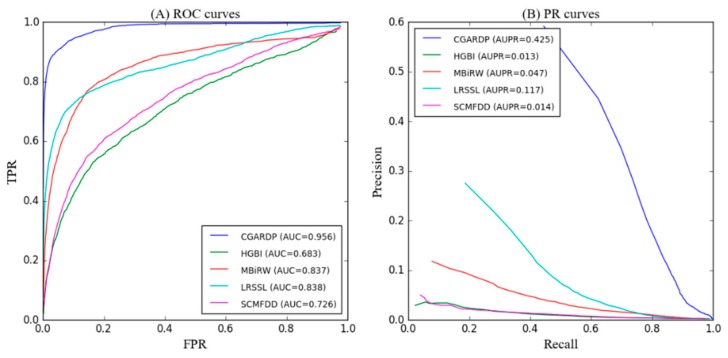
(**A**) Receiver operating characteristic (ROC) curves and (**B**) positive rate (PR) curves of CGARDP and other methods for all drugs.

As shown in Figure 4A and Table 1, CGARDP achieves the best average performance over all 763 drugs that we considered (AUC of ROC curve = 0.956). The AUC-ROC values of other methods, i.e., HGBI, MBIRW, LRSSL, and SCMFDD for 763 drugs are 0.683, 0.837, 0.838, and 0.726, respectively. In particular, CGARDP outperforms HGBI by 27.3%, MBIRW by 11.9%, LRSSL by 11.8%, and SCMFDD by 23%. Further, we list the AUCs of all five methods on 15 well characterized human drugs, each of which has more than 15 known related diseases. CGARDP yields the best average performance in terms of AUCs and achieves the best performances for 11 of the 15 common drugs. Among all methods, LRSSL performed second best, and LRSSL took full advantage of the multiple similarity of drugs. MBIRW achieved almost the same effect as LRSSL on AUC; however, it performance was less than LRSSL by 7% on AUPR. These differences in performance are possibly because MBIRW focuses on the topology information of the network. SCMFDD and HGBI perform considerably worse than LRSSL and MBIRW; however, SCMFDD performs 4.5% better than HGBI. This difference can be attributed to the fact that SCMFDD relies on the calculation of similarity, while HGBI constructs a three-layer network that introduces drug–protein information but does not make full use of this information. Compared with other methods, the superiority of CGARDP is due to its in-depth understanding of the node representation of the drug–disease association and the attentional representation of the path representation.

Because the number of unknown drug-disease associations far exceeds the known associations, there is a serious imbalance in data. The PR curve predicts performance metrics better than the ROC curve when there is a serious imbalance between the positive and negative samples. Figure 4B and Table 2 shows the AUPR for the average performance of all drugs, and CGARDP produces the best average performance on these drugs (AUC of PR curve = 0.425). Its average AUPR is 41.3%, 37.8%, 30.8%, and 41.1% higher than those of HGBI, MBIRW, LRSSL, and SCMFDD, respectively. For the 15 well-characterized drugs, CGARDP demonstrates the best performance for 11 of these drugs. In addition, 265 diseases were only association with one drug, and 116 diseases were associations with two drugs. Therefore, CGARDP can be used for diseases associated with only one or two drugs.

For all the prediction results on 763 drugs, we performed a Wilcoxon test to evaluate whether CGARDP’s performance is significantly better than that of the other methods. The statistical results (Table 3) indicate that CGARDP yields the significantly better performance under the p-value threshold of 0.05 in terms of not only AUCs but also AUPRs.

**Figure 5:**
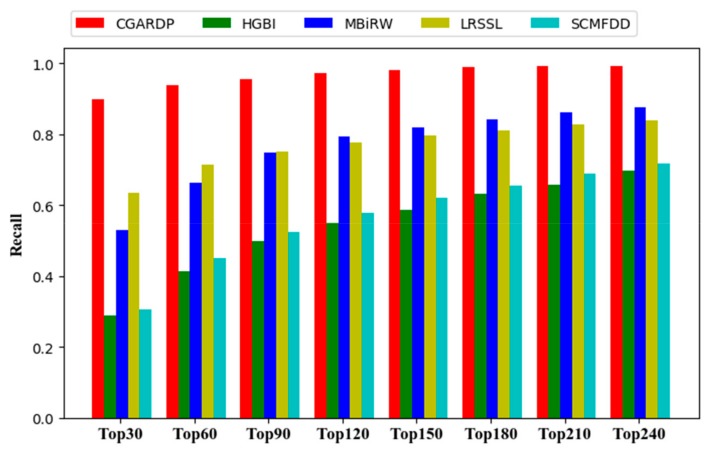
Recalls across all the tested drugs at different top *k* cutoffs.

A higher recall rate on top *k* ranked drugs means that real disease-related drugs are correctly identified. The average recall rates of the top *k* samples on all 763 drugs are shown in Figure 5. CGARDP consistently outperforms the other methods at various *k* values, and it ranked 89.9% in the top 30, 93.8% in the top 60, and 97.1% in the top 120. Before the top 90, LRSSL performed better than MBiRW, and then MBiRW surpassed LRSSL. The former ranks 63.4%, 71.3%, and 77.7% in the top 30, 60, and 120, respectively, and the latter is 53.1% and 66.3%. 79.3%. The possible reason for these different rankings is that MBiRW makes better use of global topology information, while LRSSL focuses more on neighbor node information. HGBI and SCMFDD have relatively close recall rates at different *k* values. HGBI ranks for *k* values of 30, 60, and 120 were 28.8%, 41.1%, and 54.9%, respectively, and those of SCMFDD are 30.6%, 45.0%, and 57.8%. Ultimately, we can conclude that CGARDP is indeed better than other methods in discovering the underlying disease of the drug.

### 3.3. Case Studies on Ciprofloxacin, Ceftriaxone, Ofloxacin, Ampicillin, and Levofloxacin

After the above five-fold cross-validation, we evaluated the performance of the method, and all known correlation data were used as training data to predict the unknown drug-disease association. Case studies of five drugs—Ciprofloxacin, Ceftriaxone, Ofloxacin, Ampicillin, and Levofloxacin—demonstrate the ability of CGARDP to detect high-quality candidate diseases for drugs. The analysis of each of the top ten candidates for each drug is presented in detail in Table 4.

First, A drug bank is a database of drugs pharmacology indication, drug interaction, and clinical trials for a disease. The Comparative Toxicogenomics Database (CTD) contains important information about the effects of drugs on the disease. The Centers for Disease Control and Prevention (CDC) records the trends and preventive treatments of common diseases. In Table 4, 12 candidate diseases are included from the drug bank, nine candidates are included in the CTD, and two candidates are included in the CDC; this table shows that these candidate diseases are indeed related to the corresponding drugs. Second, ClinicalTrials.gov (https://clinicaltrials.gov/) is a database of clinical trials run by the National Institutes of Health (NIH), and it contains clinical trials of various drugs and related diseases. PubChem (https://pubchem.ncbi.nlm.nih.gov/) is a database of chemical modules supported by the NIH, and it stores biochemical experimental data and structural information on compounds, including drugs and their biological activities data. A total of 21 candidate diseases in Table 4 were included in ClinicalTrials.gov, and 7 candidates were included in PubChem, indicating that these candidates were supported by the experiment. In addition, a candidate for the “literature” marker was supported by the literature. The addition of ceftriaxone to metronidazole has a synergistic effect, which can reduce the production of toxins and promote wound healing; thus, the combination of metronidazole and ceftriaxone is preventive. Tetanus patients with sepsis and pneumonia have good efficacy, confirming that Ceftriaxone affects the candidate disease tetanus.

In addition, the CTD database also contains potential associations that the literature infers to exist, labelled as Inferred. Four candidate diseases in Table 4 were inferred from the CTD literature, indicating that the drug is more likely to be associated with the candidate disease. Case studies of candidate diseases for the five drugs confirmed that CGARDP was indeed able to detect potential candidate diseases for the drug.

### 3.4. Prediction of Novel Drug–Disease Associations

According to cross validation and case studies, we applied CGARDP to predict the novel drug–disease associations. All known drug–disease associations were utilized to train CGARDP’s prediction model, the potential candidate associations were then obtained by using the model as listed in Supplementary Table S1.

## 4. Conclusions

A novel method based on CNN and GRU—CGARDP—was proposed to predict the potential drug–disease associations. The CRU based framework deeply integrates the similarity and association information of a drug–disease pair. The GGU based framework deeply learns the path information between the drug and the disease. CGARDP discriminates different contributions of the paths by constructing the attention mechanism and learns more informative representation of the drug-disease pair. The experimental results show that CGARDP outperforms other methods in terms of both AUCs and AUPRs. The case studies on five drugs confirm that CGARDP is able to retrieve potential candidate drug–disease associations.

## Figures and Tables

**Table 1:** AUCs of CGARDP and other methods for all of the drugs and 15 well characterized drugs.

Drug Name	CGARDP	HGBI	AUC MBiRW	LRSSL	SCMFDD
ampicillin	0.964	0.751	0.932	0.962	0.895
cefepime	0.990	0.910	0.970	0.971	0.914
cefotaxime	0.958	0.917	0.929	0.950	0.953
cefotetan	0.973	0.808	0.918	0.948	0.848
cefoxitin	0.880	0.890	0.912	0.979	0.894
ceftazidime	0.938	0.845	0.931	0.936	0.922
ceftizoxime	0.929	0.960	0.961	0.923	0.962
ceftriaxone	0.999	0.945	0.898	0.955	0.811
ciprofloxacin	0.905	0.811	0.813	0.928	0.820
doxorubicin	0.951	0.487	0.921	0.727	0.460
erythromycin	0.948	0.827	0.887	0.918	0.764
itraconazole	0.956	0.445	0.877	0.845	0.730
levofloxacin	0.898	0.943	0.975	0.964	0.872
moxifloxacin	0.992	0.812	0.948	0.957	0.932
ofloxacin	0.980	0.902	0.943	0.904	0.774
Average AUC	0.956	0.683	0.837	0.838	0.726

**Table 2:** AUPRs of CGARDP and other methods for all of the drugs and 15 well characterized drugs.

Drug Name	CGARDP	HGBI	AUPR MBIRW	LRSSL	SCMFDD
ampicillin	0.515	0.032	0.023	0.285	0.068
cefepime	0.766	0.163	0.315	0.625	0.054
cefotaxime	0.525	0.071	0.292	0.283	0.105
cefotetan	0.496	0.054	0.197	0.512	0.059
cefoxitin	0.420	0.151	0.394	0.286	0.065
ceftazidime	0.591	0.032	0.201	0.488	0.694
ceftizoxime	0.472	0.212	0.244	0.455	0.096
ceftriaxone	0.607	0.056	0.223	0.673	0.077
ciprofloxacin	0.429	0.082	0.118	0.280	0.064
doxorubicin	0.520	0.005	0.051	0.180	0.004
erythromycin	0.592	0.023	0.038	0.144	0.022
itraconazole	0.379	0.006	0.253	0.042	0.008
levofloxacin	0.212	0.136	0.071	0.539	0.098
moxifloxacin	0.735	0.049	0.650	0.384	0.088
ofloxacin	0.382	0.091	0.130	0.201	0.078
Average AUC	0.425	0.013	0.047	0.117	0.014

**Table 3:** The statistical result of the paired Wilcoxon test on the AUCs of 763 drugs comparing CGARDP and all of four other methods.

*p*-Value between CGARDP and Another Method	HGBI	MBiRW	LRSSL	SCMFDD
*p*-value of ROC curve	6.873 × 10^−270^	6.302 × 10^−72^	3.473 × 10^−31^	9.326 × 10^−180^
*p*-value of PR curve	4.365 × 10^−40^	7.332 × 10^−30^	2.321 × 10^−12^	3.265 × 10^−60^

**Table 4:** The top 10 candidates related to the drugs Ciprofloxacin, Ceftriaxone, Ofloxacin, Ampicillin, and Levofloxacin.

Drug Name	Rank	Disease Name	Description	Rank	Disease Name	Description
Ciprofloxacin	1	Conjunctivitis, Bacterial	Clinical Trials	6	Gram-Negative Bacterial Infections	Clinical Trials
	2	Campylobacter Infections	CDC	7	Chlamydia Infections	Clinical Trials
	3	Anthrax	CTD, Clinical Trials	8	Pneumonia, Pneumocystis	PubChem
	4	Klebsiella Infections	CTD, Clinical Trials	9	Eye Infections, Bacterial	Clinical Trials
	5	Soft Tissue Infections	Clinical Trials	10	Acanthamoeba Keratitis	PubChem
Ceftriaxone	1	Bone Diseases, Infectious	Clinical Trials	6	Tetanus	literature [38]
	2	Panic Disorder	Drug Bank	7	Legionnaires Disease	Drug Bank
	3	Hepatitis B	Clinical Trials	8	Cytomegalovirus Infections	Drug Bank
	4	Respiratory Syncytial Virus Infections	PubChem	9	Respiration Disorders	Clinical Trials
	5	Maxillary Sinusitis	Drug Bank	10	Respiratory Distress Syndrome, Adult	Clinical Trials
Ofloxacin	1	Corneal Ulcer	PubChem	6	Proteus Infections	CTD
	2	Epididymitis	CDC	7	Urinary Bladder Neck Obstruction	Inferred candidate by 1 literature
	3	Otitis Externa	Drug Bank	8	Glaucoma, Angle-Closure	PubChem
	4	Tuberculosis, Pulmonary	CTD, clinical Trials	9	Urinary Bladder Diseases	Inferred candidate by 1 literature
	5	Urethral Diseases	PubChem	10	Trichomonas Vaginitis	clinical Trials
Ampicillin	1	Burns	Inferred candidate by 3 literature	6	Candidiasis, Cutaneous	PubChem
	2	Meningitis, Bacterial	CTD	7	Otitis Media, Suppurative	Drug Bank
	3	Pseudomonas Infections	CTD	8	Pneumonia, Bacterial	CTD, Clinical Trials
	4	Skin Diseases, Infectious	Clinical Trials	9	Proteus Infections	CTD
	5	Radiation Injuries, Experimental	Inferred candidate by 1 literature	10	Sarcoma, Ewings	Drug Bank
Levofloxacin	1	Tuberculosis, Pulmonary	Clinical Trials	6	Listeriosis	Drug Bank
	2	Histoplasmosis	Drug Bank	7	Soft Tissue Infections	CTD, Clinical Trials
	3	Pneumonia, Mycoplasma	Clinical Trials	8	Respiratory Tract Fistula	Drug Bank
	4	Bronchitis	Clinical Trials	9	Rhinitis	Drug Bank
	5	AIDS-Related Opportunistic Infections	Clinical Trials	10	Mouth Diseases	Clinical Trials

## Supplementary Materials

The following are available online, Table S1: The top 10 potential candidates for 763 drugs.
